# On the Progress of Medicine

**Published:** 1804-11-01

**Authors:** David Uwins

**Affiliations:** Somers Place, St. Pancras


### On the Progress of Medicine; *communicated by* David Uwins, M. D. *Somers Place, St. Pancras.*

"Vis consili expers mole ruit suâ."

THE rapid progress which science has made within the few past years has exceeded calculation; a disposition to the acquisition of knowledge has pervaded every class and all ranks of society. Every one, in the present day, who is not entirely devoted to sensual gratification, or totally destitute of what alone gives dignity to the human character, and marks the superiority of man; every one is stimulated by the ardour of enquiry, and impelled to the cultivation of his intellectual powers. Philosophy is no longer concealed by the walls of a college, or its advantages and pleasures enjoyed by a chosen few; but, deprived of its former severity, and stripped of its imposing garb, it at length stands revealed to public view; its benign influence is generally experienced, and universally acknowledged.

While, however, we indulge the pleasure arising from the consciousness of the rapid advancement of general science, we cannot but regret that the science of medicine in its progress is comparatively tardy, and that its advancement is by no means proportionate to that of other branches of knowledge. A general enquiry, therefore, into the cause of this, may not, perhaps, prove entirely nugatory.

That that department of knowledge, compared to which, did it accomplish its object, all other sciences were vain and futile, and which must ever be considered as the noblest pursuit which can engage the mind or exercise the talents of man; that a science so grand in its nature and important in its design, should continue almost stationary, while others of less utility and inferior value are found to advance with astonishing rapidity, must occasion surprise and awaken enquiry.

It cannot be denied that the progress of the medical in common with that of all other sciences, has been considerably retarded by the general obstructions to liberal pursuits, superstition, and credulity; but now that the

 veil veil of mystery is removed, that the reasoning faculty is exercised more than the senses and imagination, that an universal disposition to impartial enquiry has obtained, it appears that some cause, operating peculiarly to the retardment of this department of knowledge, must be suspected and sought for: and it is, in my opinion, not to be attributed to the undemonstrable nature of the science itself, but to the mode of instruction which is still adopted in the schools and by the professors of medicine.

While in every other branch of education the inductive method of reasoning and instruction has so generally obtained, and in which, hypotheses are always timidly advanced, and published rather as casual suggestions than doctrines requiring belief and attention, Professors being fully aware that those principles alone deserve the appellation of science which stand on the firm basis of facts and experiment;—in medicine, on the contrary, "the *high a priori road*" is still pursued; and, as if conscious of a deficiency in true knowledge, our instructions in this department of education, at least the generality of them, amuse the fancy and exercise the imagination by the promulgation of doctrines, and the formation of systems, for which they have not the authority of either nature or experience.

Professors of the healing art appear to indulge a species of self-deception; and conscious at the same time of the importance of their science, and its imperfect state, so far as demonstrative evidence is required, they endeavour to remedy this imperfection by the substitution of splendid and fanciful hypotheses, by generalizing without facts, and drawing conclusions without data.

Why this disposition to illegitimate generalization should continue in medicine, while it is expunged from other branches of philosophy, I must confess myself at a loss to explain, unless it arise, as I have above hinted, from a consciousness of paucity of real and useful information.

Such vanity and determination, at all events, to support the dignity and splendour of our science, were, perhaps, in some measure excusable, and would not so loudly call for deprecation and censure, were it not positively, as well as negatively, injurious to the interests of society, and greatly obstructive to the advancement of the art: for while dogmas are substituted for facts, and systems for science, our progress in improvement must necessarily be slow.

Let

Let us then no longer deceive ourselves;—let us cease fondly to imagine, that while engaging in endless disputations about unknown principles and imaginary qualities, we *can* be in a state of progressive improvement, or add to the stock of useful knowledge.

The logical positions and metaphysical discussions of the antient schoolmen, are now only mentioned to excite a smile. Reason has at length assumed her empire over the mind of man, and has conducted him to the direct and only safe road of experimental enquiry: Let us then, where it is most required, follow her steps, and be guided by her dictates; extricate ourselves from the trammels of imaginary knowledge, and maintain the dignity of the science of medicine by other than artificial methods. Then, and not till then, will our exertions be crowned by abundant success; then will scepticism be banished from physic, and we shall be enabled, on proper grounds, to assert our claim to public attention and regard.

I shall endeavour to pursue this subject in a future letter, if the Editors of the Medical and Physical Journal do me the honour of publishing the present; till then,

I am, &c.

*August* 21, 1804. DAVID UWINS, M.D.

